# Editorial Comment from Dr Yuasa to Possible abscopal effect after discontinuation of nivolumab in metastatic renal cell carcinoma

**DOI:** 10.1002/iju5.12199

**Published:** 2020-07-27

**Authors:** Takeshi Yuasa

**Affiliations:** ^1^ Department of Urology Cancer Institute Hospital Japanese Foundation for Cancer Research Tokyo Japan

Human programmed death‐1 immune checkpoint inhibitor therapy is being rapidly introduced in metastatic renal cell carcinoma (mRCC) clinical practice in Japan, dramatically changing the therapeutic strategy for mRCC. The combination of radiation and immune checkpoint inhibitor therapies has gained particular interest because many reports have indicated that, in addition to radiation‐targeted lesions, non‐targeted distant lesions could also be shrunk by simultaneous or sequential combinations.^1^ Although the precise mechanism of the abscopal effect remains unclear, an increasing number of reports in the current immune checkpoint inhibitor era support the hypothesis that activation of the immune system as well as modulation of the tumor microenvironment by radiation and immune checkpoint inhibitor therapies definitely play important roles in the abscopal effect.^2^

In this issue of *IJU Case Reports*, Nakajima *et al*. reported a possible abscopal effect of radiation therapy after discontinuation of nivolumab in mRCC.^3^ In this report, a patient with mRCC of International mRCC Database Consortium intermediate risk underwent targeted therapy using pazopanib, axitinib, and everolimus. Consequently, nivolumab was administered as fourth‐line therapy.^3^ A right iliac bone metastasis that appeared after the completion of nivolumab therapy was treated with radiation therapy (30 Gy, 10 fractions).^3^ Five months after radiotherapy, significant reductions were observed in multiple metastases (lung, right kidney, and subcutaneous tissue). Nine months after radiotherapy, no progression was noted even though no additional systemic therapy was administered.^3^

RCC has historically been considered intrinsically radioresistant, and the radioresistance has been verified through *in vitro* experiments, which demonstrated that the RCC cell line was amongst the most radioresistant.^4^ Very recently, however, at the 2020 American Society of Clinical Oncology Genitourinary Cancers Symposium, two phase II trials evaluating the combination of immune and radiation therapies in mRCC were presented. The RADVAX RCC study evaluated the combination of nivolumab and ipilimumab with stereotactic body radiation therapy (50 Gy, 5 fractions).^5^ The objective response rate, median progression‐free survival (PFS), and 1‐year PFS rate were 56%, 8.21 months, and 36%, respectively.^5^ Various other clinical trials which attempt to clarify the efficacy and safety of the combination of immune and radiation therapies are underway. Once the precise mechanism and the optimal radiation characteristics have been determined, the abscopal effect may be incorporated into treatment strategies as an important option in mRCC.

## Conflict of interest

The author declares no conflict of interest.
